# Compressing and expanding optical matrix-vector multipliers for enabling optical image encoder-decoders and generators

**DOI:** 10.1038/s41377-025-02141-0

**Published:** 2026-01-03

**Authors:** Adrian Stern

**Affiliations:** Department of Electro-optics and Photonics Engineering, School of Electrical and Computer Engineering, Ben-Gurion University of the Negev, Beer-Sheva, Israel

**Keywords:** Optical manipulation and tweezers, Imaging and sensing

## Abstract

Both compressing and expanding optical matrix-vector multipliers are necessary for the full optical realization of neural networks. An expanding multiplier scheme is proposed, which, together with common compressing multipliers, is employed to demonstrate image processor networks such as autoencoders and image generators.

Matrix-Vector Multiplications (MVMs) are at the core of any Deep Neural Network (DNNs), determining the data flow and the execution of computations. Figure 1a. shows a fully-connected neural network layer realizing an MVM (Fig. 1b) followed by a nonlinear activation function. Mathematically, the layer’s operation is expressed as ${\bf{y}}=f\left({\bf{W}}\cdot {\bf{x}}\right)$ where ${\bf{x}}\in {{\mathbb{R}}}^{K}$, ${\boldsymbol{y}}\in {{\mathbb{R}}}^{N}$, ${\bf{W}}\in {{\mathbb{R}}}^{K\times N}$ and *f* is a nonlinear activation function.

**Fig. 1:**
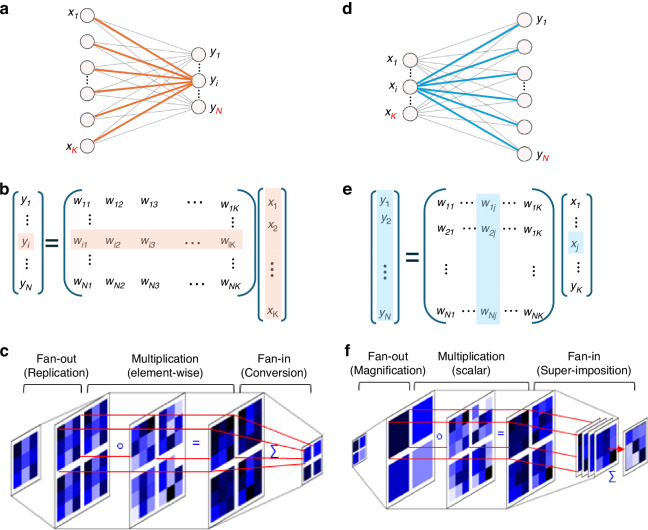
Compressing and expanding optical matrix-vector multiplication. **a** Schematic of a fully-connected DNN layer performing a contracting (Type 1) MVM operation (**b**). The red-shaded components in (**b**) highlight the *i*’th vector dot product corresponding to the red lines in (**a**). **c** Schematics of Type 1 MVM realization for *K* = 9 and *N* = 4, involving image replication, element-wise multiplication by a weight mask, and a fan-in. **d**–**f** fully connected NN layer performing expanding (Type 2) MVM operation. The blue shaded components in (b) correspond to the blue lines in (**a**). **e** Schematic of Type 2 MVM realization for *K* = 4, *N* = 9, involving image magnification, scalar multiplication of the weight mask, and a fan-in. After ref. ^15^

The rate-limiting step in DNN inference is the MVM operations. MVMs are the most computationally intensive operations, particularly in fully-connected (dense) layers and attention mechanisms. Traditional digital implementations of these operations face challenges such as high energy consumption and limitations in parallelism and speed. In MVMs, the most resource-intensive part involves transferring weight and input data from memory to the multiplier units, which is essential for the multiply-accumulate (MAC) operation. More recent DNN accelerators, that utilize weight-stationary architectures, still encounter trade-offs related to latency, power consumption, and scalability^1^.

The recognition that electronic MVM remains a notable computational bottleneck, despite advancements in digital hardware like graphics and tensor processors, has sparked renewed interest in the optical realization of MVMs. Optics has long been recognized as a promising medium for matrix multiplication and interconnections due to its low power consumption, low latency, and the potential for parallel processing^2^. Optical MVMs were pioneered nearly half a century ago with the introduction of the “Stanford MVM” by Goodman et al.^3^. They have since been applied in neural networks, starting with the seminal work of Farhart et al.^4^. In recent years, there has been a renewed interest in developing optical processors due to the rapid evolution of artificial intelligence, accompanied by its huge computing demands. Various methods for optical and photonic MVM have been developed (see, for example, refs. ^5,6^). Among them, 3D optical MVMs are recognized for the high connectivity between potentially millions of spatial modes^1,7^.

Current optical neural networks (ONNs) systems have shown superior performance in encoding operation, using optical MVMs to compress image data before digital conversion^1,8,9^. Encoding generally involves dimensional reduction, which necessitates the use of compressing (aka contacting) MVMs, such that $K > N$. For incoherent imaging purposes, 3D optical MVMs are commonly realized by passing the input image through multiple masks, each mask representing a row in **W**^1,4,7,10–14^. In a recently published paper in *Light: Science & Applications*^15^, this kind of MVMs were dubbed as Type 1 multipliers. Their operation involves performing parallel vector-vector dot products between the input vector and weight vectors, realizing ${y}_{i}={{\bf{W}}}_{i,:}{\bf{x}},{i}=1,..N$, where ${{\bf{W}}}_{i,:}\in {{\mathbb{R}}}^{K}$ represents the *i*’th row of the matrix **W**. This inner product is illustrated by the shaded components in the matrix **W** in Fig. 1b. The weights of ${{\bf{W}}}_{i,:}$ are highlighted by red lines in the fully-connected layer depicted in Fig. 1a. These weights constitute the mask elements in the optical schematics shown in Fig. 1c, realized with an LCD in ref. ^15^. In general, the optical implementation complexity is determined by the number of masks, which is determined by the number of rows of **W**. Thus, for encoding operations, where $K > N$, Type 1 scheme is an appropriate choice.

However, for ONN implementing autoencoders, variational autoencoders, U-Net, attention blocks, and generative architectures, decoding operations are required. Decoding operations necessitate MVM with output layers that are larger than the input layers, that is, ${K} < N$. For this setting, Type 2 MVM was proposed in the newly published paper in *Light: Science & Applications*^15^. The design of Type 2 optical MVM relies on the observation that the MVM can be alternatively expressed as ${\bf{y}}={\sum }_{j=1}^{K}{x}_{j}{{\bf{W}}}_{:,j}$ where ${{\bf{W}}}_{:,j}$ is the *j*-th column of the matrix **W** is (as illustrated in Fig. 1e). Mathematically, Type 1 MVM can be viewed as the application of **W** on **x** to produce **y**, while Type 2 MVM can be interpreted as **x** acting on **W** to produce **y**. Each column of the matrix **W** is realized by a coded mask; thus, a total of $K( < N)$ masks are required for the scheme in Fig. 1e.

In ref. ^15^, optical encoders based on Type 1 MVM and decoders utilizing Type 2 MVM were employed to demonstrate several DNN models. The dimensionality-increasing property of Type 2 MVM enabled the realization of regression-type DNNs, that produce images as output, unlike most previous fully incoherent ONNs, which were limited to classification tasks. The authors in ref. ^15^ demonstrated an ONN autoencoder that utilizes a Type 1 multiplier for encoding *K* = 28 × 28 pixels monochromatic images from the MNIST, Fashion-MNIST, and K-MNIST datasets into a bottleneck with a dimension of *N* = 18. For the decoding process, they employed two parallel Type 2 matrix-vector multiplications (MVMs): one for positive elements and another for negative elements of the weight matrix **W**. The outputs of these two operations were then summed up and activated electronically. To mitigate hardware imperfections and noise, an on-system iterative progressive calibration process was developed, yielding image reconstruction accuracy comparable to that of a digital autoencoder. Compared to previously presented diffractive autoencoders^16^, the autoencoder in ref. ^15^ is not limited to coherent illumination and has a distinct bottleneck. Furthermore, the autoencoder was trained to function as a denoising DNN, demonstrating its capability to effectively remove noise from images with a peak signal-to-noise ratio (PSNR) as low as 8 dB.

Type 2 MVMs are crucial for developing optical image generator models, which rely on expanding networks to create new images from a latent space. Reference ^15^ demonstrated the effectiveness of these optical decoders for variational autoencoders (VAEs) and generative adversarial networks (GANs). As essential enabling technology for ONNs, Type 2 matrix-vector multipliers (MVMs) hold a great promise for powering more advanced, multilayered^8,13^, ultrafast, and power-efficient optical image processors.
